# Neuropeptide S (NPS) variants modify the signaling and risk effects of NPS Receptor 1 (NPSR1) variants in asthma

**DOI:** 10.1371/journal.pone.0176568

**Published:** 2017-05-02

**Authors:** Nathalie Acevedo, Sini Ezer, Simon Kebede Merid, Vincent D. Gaertner, Cilla Söderhäll, Mauro D’Amato, Michael Kabesch, Erik Melén, Juha Kere, Ville Pulkkinen

**Affiliations:** 1 Department of Clinical Science and Education, Karolinska Institutet, and Sachs' Children and Youth Hospital, Södersjukhuset, Stockholm, Sweden; 2 Research Programs Unit, Program for Molecular Neurology, University of Helsinki, and Folkhälsan Institute of Genetics, Helsinki, Finland; 3 Institute of Environmental Medicine, Karolinska Institutet, Stockholm, Sweden; 4 Department of Pediatric Pneumology and Allergy, University Children's Hospital Regensburg (KUNO), Regensburg, Germany; 5 Department of Biosciences and Nutrition, Karolinska Institutet, Huddinge, Sweden; 6 Department of Women´s and Children´s Health, Karolinska Institutet, Stockholm, Sweden; 7 BioDonostia Health Research Institute and IKERBASQUE, Basque Foundation for Science, San Sebastian Spain; 8 Clinical Epidemiology Unit, Department of Medicine Solna, Karolinska Institutet, Stockholm, Sweden; 9 Sachs’ Children’s Hospital, Stockholm, Sweden; 10 Heart and Lung Center, Division of Pulmonary Medicine, University of Helsinki and Helsinki University Hospital, 00014 University of Helsinki, Helsinki, Finland; National Taiwan University College of Public Health, TAIWAN

## Abstract

Single nucleotide polymorphisms (SNPs) close to the gain-of-function substitution, Asn(107)Ile (rs324981, A>T), in Neuropeptide S Receptor 1 (*NPSR1*) have been associated with asthma. Furthermore, a functional SNP (rs4751440, G>C) in Neuropeptide S *(NPS)* encodes a Val(6)Leu substitution on the mature peptide that results in reduced bioactivity. We sought to examine the effects of different combinations of these *NPS* and *NPSR1* variants on downstream signaling and genetic risk of asthma. In transfected cells, the magnitude of NPSR1-induced activation of cAMP/PKA signal transduction pathways and downstream gene expression was dependent on the combination of the NPS and NPSR1 variants with NPS-Val(6)/NPSR1-Ile(107) resulting in strongest and NPS-Leu(6)/NPSR1-Asn(107) in weakest effects, respectively. One or two copies of the NPS-Leu(6) (rs4751440) were associated with physician-diagnosed childhood asthma (OR: 0.67, 95%CI 0.49–0.92, p = 0.01) and together with two other linked *NPS* variants (rs1931704 and rs10830123) formed a protective haplotype (p = 0.008) in the Swedish birth cohort BAMSE (2033 children). *NPS* rs10830123 showed epistasis with *NPSR1* rs324981 encoding Asn(107)Ile (p = 0.009) in BAMSE and with the linked *NPSR1* rs17199659 (p = 0.005) in the German MAGIC/ISAAC II cohort (1454 children). In conclusion, *NPS* variants modify asthma risk and should be considered in genetic association studies of *NPSR1* with asthma and other complex diseases.

## Introduction

Neuropeptide S (NPS) affects multiple neuroendocrine, behavioral, and inflammatory responses via its G protein-coupled cell surface receptor NPSR1 (Neuropeptide S Receptor 1) [1–4]. *NPSR1* was identified as a susceptibility gene for asthma and related traits by positional cloning and the associations of *NPSR1* single nucleotide polymorphisms (SNPs) with asthma have been replicated in ethnically diverse populations [5–12], and marginally supported by a large-scale genome-wide association study (GWAS) [13]. In addition, *NPSR1* SNPs have shown genetic associations with other inflammatory phenotypes such as inflammatory bowel disease [14] and rheumatoid arthritis [15, 16]. However, the *NPSR1* locus has shown allelic heterogeneity with different markers (tag SNPs, intronic markers, and haplotypes) showing associations depending on the study design and population being studied. We have previously shown that several susceptibility alleles of low-to-moderate-effects in *NPSR1* may modify the asthma risk and show epistasis depending on the carrier status for variants in genes belonging to common biological pathways [17]. These effects may not be uncovered by SNP arrays which are suitable for detection of common polymorphisms but cannot detect the effects of less frequent coding mutations and low frequency functional SNPs.

The functional *NPSR1* SNP rs324981 (A>T), encoding a substitution of Asn(107)Ile in the putative ligand-binding pocket of NPSR1 [5], has shown associations with neuropsychiatric phenotypes, such as panic disorders [18–20], psychological stress [21], and fear responses [22, 23]. A GWAS on circadian sleep parameters found an association between rs324981 and regular bedtime [24] and another study showed that the same SNP was associated with sleep and rest duration [25]. In cell models, the change of Asn(107) to Ile(107) results in 10-fold increase in NPS-mediated intracellular signaling [26] and changes in genome-wide transcriptional profiles [27]. In addition, the *NPSR1* SNP rs324981 has been associated with airway hyperresponsiveness to methacholine in a Chinese population [8]. Non-coding functional SNPs have also been detected in *NPSR1*, for instance rs2530547 which affects luciferase expression in gene reporter assays and *NPSR1* mRNA levels in human leukocytes [27]. By changing the levels of expression in this receptor, they may ultimately also affect signaling, and are hypothesized to affect neuroinflammatory phenotypes [28]. Thereby associations with individual *NPSR1* SNPs needs to be evaluated in the context of gene-gene interactions because a combination of functional polymorphisms may ultimately determine receptor properties and/or expression levels [27].

A functional SNP rs4751440 in *NPS* encodes a Val(6)Leu substitution in the mature NPS peptide that results in lower bioactivity [29]. We hypothesized that the interaction of functional *NPS* and *NPSR1* SNPs might lead to either strong or minimal downstream signaling depending on the genotype combination on both loci. The aims of this study were: 1) To clarify the potential functional crosstalk between NPS and NPSR1 variants; 2) to identify the impact of putative *NPS* risk alleles on the susceptibility to asthma; and 3) to evaluate potential gene-gene interaction effects (epistasis) between *NPS* and *NPSR1* polymorphisms on asthma.

## Results

### Biological interaction of NPS and NPSR1 variants can lead to either strong or minimal downstream signaling

**Fig 1:**
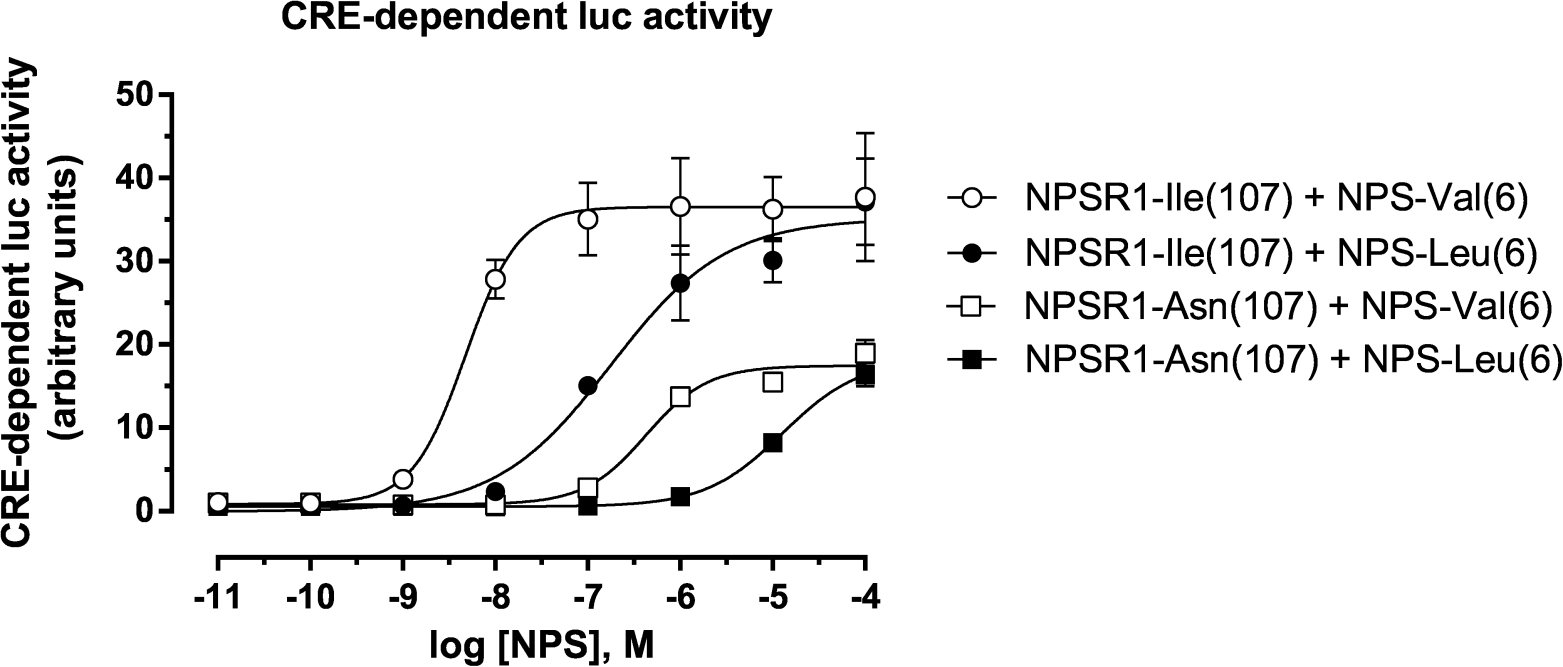
NPS variants and CRE luciferase activity. Dose-response curves of NPS-Val(6) and NPS-Leu(6) (10 pM– 100 μM) based on the cAMP response element (CRE) luciferase activity in human embryonic kidney epithelial (HEK293) cells transiently transfected with either NPSR1-Ile(107) or NPSR1-Asn(107) coding variants. One representative experiment is shown. The CRE-driven luciferase activity is expressed in mean arbitrary units ± SEM of triplicates 3 h after NPS stimulation.

NPS is known to activate NPSR1 by increasing cAMP production [30, 31]. To compare the bioactivities of the two NPS variants, we measured the dose-responses of NPSR1-Ile(107) and NPSR1-Asn(107) coding variants to either wildtype NPS-Val(6) or alternative NPS-Leu(6) in a luciferase assay dependent on the activation of a cAMP-response element (CRE). This assay was used since elevation of the intracellular cAMP levels activates cAMP response element binding protein (CREB) via phosphorylation of protein kinase A (PKA). Activated CREB binds to CRE and induces the expression of downstream target genes of the cAMP/PKA signaling pathway. Because of the low endogenous expression levels of NPSR1 in majority of the available cell lines [26, 29], we first used HEK293 cells with forced NPSR1 expression as a model for ligand-receptor interactions. This approach allowed comparisons between the present and previous studies [26, 29]. HEK293 cells were transiently co-transfected with the two NPSR1 variants along with a CRE-responsive firefly luciferase construct and a construct constitutively expressing Renilla luciferase. Firefly and Renilla luciferase activity was measured 3 h after stimulation with serial dilutions of NPS (10 pM-100 μM). As shown in **Fig 1**, wildtype NPS—Val(6) peptide stimulated CRE—dependent luciferase activity at 50—fold lower concentrations than the NPS—Leu(6) peptide in NPSR1—Ile(107) cells (pEC 50: 8.4 ± 0.2 and 6.7 ± 0.2, p = 0.004, respectively), and at 15-fold lower concentrations in NPSR1—Asn(107) cells (pEC 50: 6.1 ± 0.3 and 4.9 ± 0.1, p = 0.03, respectively).

The maximum of luciferase activity was ~2-fold in cells transfected with the NPSR1-Ile(107) variant in comparison to the cells transfected with the NPSR1-Asn(107). Thus, the magnitude of NPSR1-induced activation of CRE was markedly different depending on the combination of the NPS and the NPSR1 variants resulting in either strong (NPS-Val(6) and NPSR1-Ile(107)) or minimal (NPS-Leu(6) and NPSR1-Asn(107)) downstream signal transduction.

**Fig 2:**
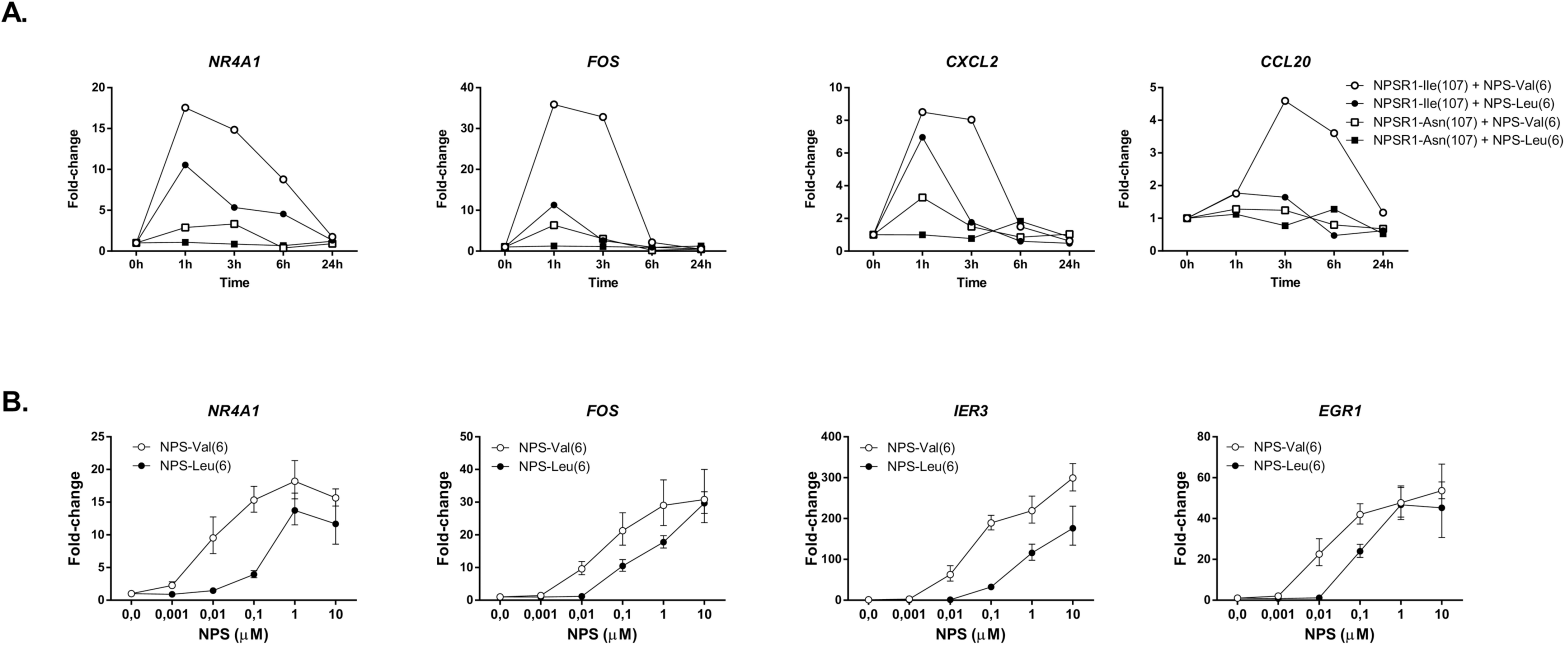
Real time RT-PCR analysis of NPSR1 downstream target genes. (A) Time-dependent mRNA expression of *NR4A1*, *FOS*, *CXCL2*, and *CCL20* in human embryonic kidney epithelial (HEK293) cells transiently transfected with either NPSR1-Ile(107) or NPSR1-Asn(107) coding variants and stimulated with either NPS-Val(6) or NPS-Leu(6) NPS (100 nM) for 1, 3, 6, and 24 h. (B) Dose-response curves of *NR4A1*, *FOS*, *IER3*, and *EGR1* in human SH-SY5Y stable cell line over-expressing NPSR1-Ile(107) stimulated with either NPS-Leu(6) or NPS V6 (0.0001–1 μM) for 3 h. The results are presented as fold-changes in comparison to the unstimulated cells. *GAPDH* was used as the endogenous reference, and data are expressed as mean of triplicate samples. The error bars represent 95% confidence intervals. In all experiments, results were calculated with the comparative *C*_*t*_ method.

The activation of CRE results in transcriptional changes of downstream target genes. To examine whether the observed differences in the promoter activity also affected gene expression, we measured the effects of NPS variants on known NPSR1 downstream target genes [27, 32, 33]. *NR4A1* (nuclear receptor subfamily 4, group A, member 1), *FOS* (FBJ murine osteosarcoma viral oncogene homolog), and chemokine ligands *CXCL2* and *CCL20* were selected as they were among the most influenced genes after NPS stimulation and their expression also differed between transcriptome comparisons of HEK293 cells expressing either NPSR1-Ile(107) or NPSR1-Asn(107) [27]. In the present study, HEK293 cells transiently transfected with either NPSR1-Ile(107) or NPSR1-Asn(107) coding variants were stimulated with 100 nM of either NPS-Val(6) or NPS-Leu(6) for 1, 3, 6, and 24 h, and changes in gene expression were analyzed with RT-PCR. In agreement with the results from the luciferase assay, NPS induced stronger downstream signaling in cells expressing NPSR1-Ile(107) than in cells expressing NPSR1-Asn(107), and wildtype NPS-Val(6) peptide was more potent than the NPS-Leu(6) peptide. mRNA expression of *NR4A1*, *FOS*, and *CXCL2* was induced 1 h after NPS stimulation whereas the expression of *CCL20* was up-regulated at 3 h (**Fig 2A**).

Altered neuroinflammatory mechanisms have been implicated in allergic airway inflammation [34]. Because NPSR1 is expressed in neuroendocrine tissues [35–37], we used SH-SY5Y neuroblastoma cells of neuroendocrine origin to validate the changes in gene expression observed in HEK293 cells. SH-SY5Y cells stably overexpressing NPSR1-Ile(107) [17, 36] were stimulated with either NPS-Leu(6) or NPS-Val(6) (0.0001–1 μM) for 3 h and the mRNA expression of known downstream target genes in these cells [36] was detected using RT-PCR. As expected, NPS stimulation increased the expression of *NR4A1*, *FOS*, *EGR1* (early growth response protein 1), and *IER3* (immediate early response 3) in SH-SY5Y cells over-expressing NPSR1-Ile(107) in a dose dependent manner (**Fig 2B**). Wildtype NPS-Val(6) peptide was more potent than the NPS-Leu(6) peptide. These observations validated the results detected in HEK293 cells in an independent cell line.

### *NPS* rs4751440 encoding Leu(6) is protective for childhood asthma

**Fig 3:**
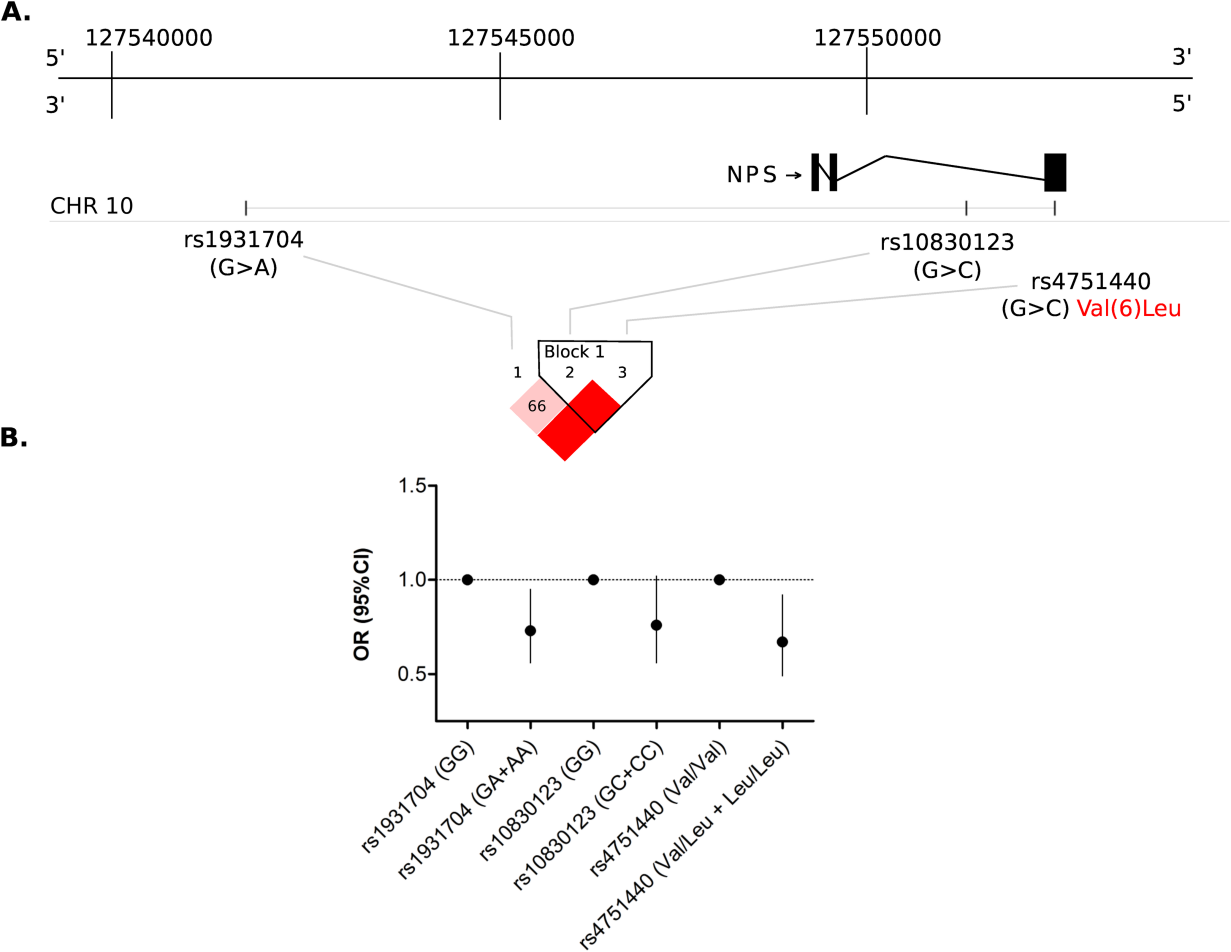
Location and block structure of three genotyped SNPs in the *NPS* gene. (A) The scale represents the relative positions of rs1931704 (G>A) in the upstream regulatory region of *NPS*, followed by rs10830123 (G>C) in the intronic region and rs4751440 (G>C) in the coding region. Allele changes are indicated by > and given on the positive chromosomal strand of the Human Reference Assembly. The arrow indicates *NPS* transcription from the positive strand. (B) Effects of *NPS* SNPs on the risk of physician-diagnosed asthma in carriers of either one or two copies of the alternative allele. Bars represent 95% confidence intervals (CI). OR: Odds Ratio. Given the allele and genotype frequencies, the dominant model which best fits the data is shown.

Because the SNP rs4751440 (G/C) encoding for NPS-Val(6) and NPS-Leu(6) is common in European and American mixed populations [29], and given its functional effects on NPSR1 signaling in cell assays, we hypothesized that this SNP might influence the genetic risk of diverse disease traits including asthma. The rs4751440 Val(6)Leu and two other *NPS* SNPs (rs1931704 and rs10830123) were analyzed for associations with physician-diagnosed childhood asthma in the prospective Swedish birth-cohort BAMSE (n = 2033) [38, 39]. The SNPs spanned a region of 11 kb on chromosome 10q26.2 and were in linkage disequilibrium (LD) with each other **(Fig 3A)**. The allele frequencies of the three *NPS* SNPs in cases and controls and the results of the association tests with asthma are presented in **S1 Table**. Two *NPS* SNPs (rs1931704 and rs4751440) in strong linkage disequilibrium (D’ = 0.99, r^2^ = 0.69) were significantly associated with physician-diagnosed childhood asthma at age 8 years under dominant (p = 0.01) and additive models (p = 0.03). For both *NPS* SNPs the effect was driven by a higher frequency of the minor allele in the control group (**Fig 3B**). These results revealed that the allele C in *NPS* rs4751440 encoding for Leu(6) is protective for asthma. The association between *NPS* variants and asthma was also significant when comparing the haplotype frequencies between asthmatic children and controls with a protective effect driven by the haplotype AGC (p = 0.008) (**Table 1**). There were no significant associations between *NPS* SNPs and atopic sensitization (data not shown).

**Table 1:** Haplotype association of NPS polymorphisms with asthma at 8 years.

Haplotype	rs1931704 (G>A)	rs10830123 (G>C)	rs4751440 (G>C)	Frequency (controls)	Frequency (asthmatics)	OR 95%CI	(p-value)
1	G	G	G	0.70	0.76	1.0	-
**2**	**A**	**G**	**C**	**0.14**	**0.10**	**0.71 (0.53–0.94)**	**0.01**
3	A	C	G	0.11	0.10	0.87 (0.65–1.16)	0.3

Global haplotype association **p-value = 0.0077**

Allele changes are indicated by > and given on the positive chromosomal strand of the Human Reference Assembly.

### Gene-gene interactions between *NPS* and *NPSR1* variants

**Fig 4:**
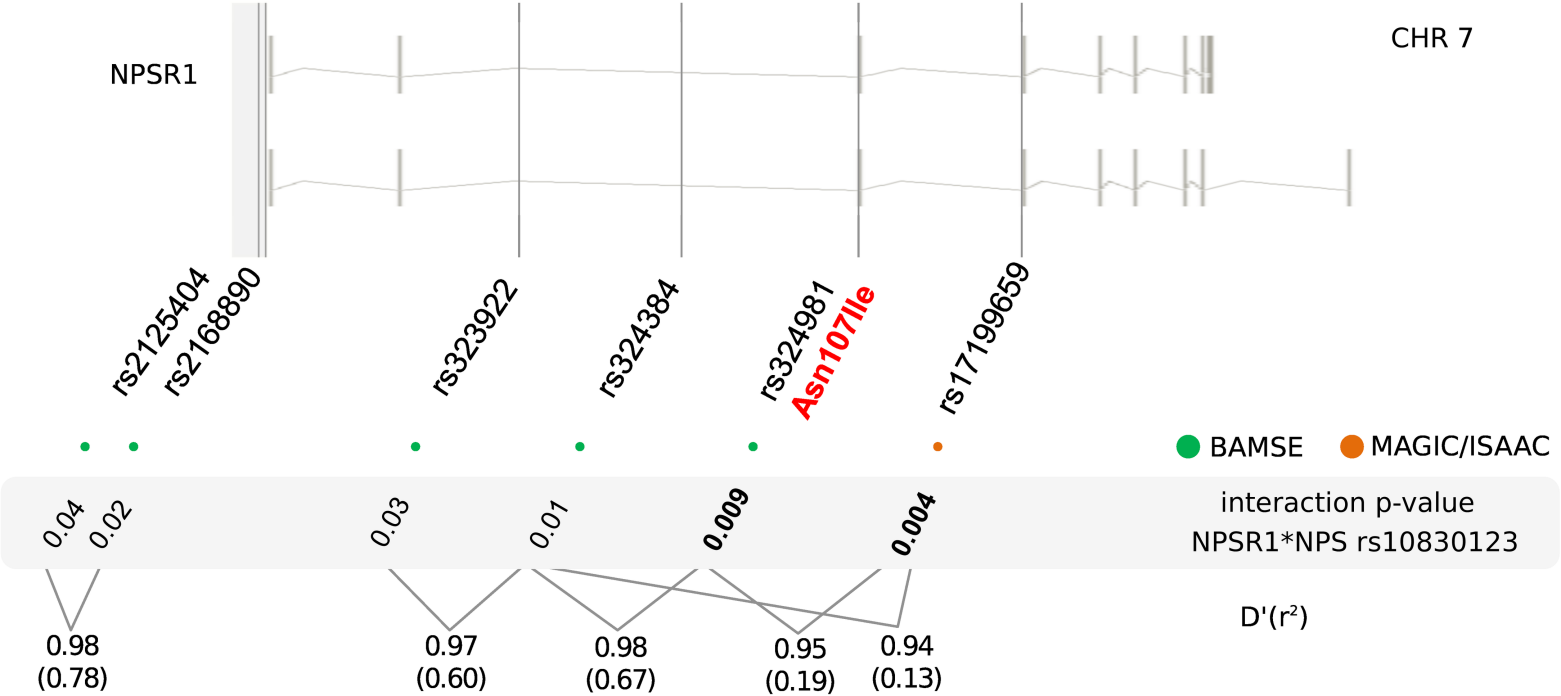
Gene-gene interactions between NPSR1 and NPS. Location of six *NPSR1* SNPs with significant interactions with *NPS* rs10830123 in the multiplicative model for epistasis in connection with their p-value for interaction and linkage disequilibrium. The most significant interaction signals in BAMSE (green) and MAGIC/ISAAC (orange) are marked in bold. Statistics on LD between the functional rs324981 and other interacting SNPs are provided as D’ and r^2^.

Based on the effects of the *NPS* SNP rs4751440 on genetic asthma risk and NPSR1 signaling, we first tested genetic association of 29 *NPSR1* SNPs with physician-diagnosed childhood asthma in BAMSE (n = 2033), and then assessed epistasis between *NPS* and *NPSR1*. We detected significant association between three *NPSR1* SNPs (rs887020, rs2531840 and rs11770777) with asthma (p<0.05) and confirmed that the functional rs324981 (T>A) encoding for NPSR1-Ile(107) and NPSR1-Asn(107) had no significant main effects at present resolution **(S2 Table)**. The next aim was to investigate if the effects of *NPS* SNPs (rs1931704, rs10830123 and rs4751440) on the risk of asthma were modified by the carrier status of alleles in the SNP rs324981 (A>T) encoding for NPSR1-Asn(107) and NPSR1-Ile(107), respectively, or by other variants with previous evidence of putative functional effects in the *NPSR1* gene. We detected significant interaction between *NPS* SNP rs10830123 and *NPSR1* rs324981 (interaction p value = 0.009). There were also significant interactions between *NPS* SNP rs10830123 with two additional *NPSR1* SNPs (rs323922 and rs324384) in linkage disequilibrium with *NPSR1* rs324981 **(Fig 4)**. A second region with significant interactions between *NPS* and *NPSR1* was detected between two SNPs in the *NPSR1* promoter (rs2125404 and rs2168890) and *NPS* SNP rs10830123 (**Fig 4**) and **S3 Table**.

We then explored the gene-gene interaction between *NPS* and *NPSR1* SNPs in the MAGIC/ISAAC cohort (n = 1454). *NPS* and *NPSR1* SNPs did not show any significant main effects on asthma risk in this dataset (data not shown). However, there was a significant interaction between *NPS* rs10830123 and *NPSR1* rs17199659 (p = 0.005) (**Fig 4 and S3 Table**). *NPSR1* rs17199659 is in LD (D’ = 0.95, r^2^ = 0.19) with *NPSR1* rs324981 encoding for NPSR1-Asn(107) and NPSR1-Ile(107) and with rs324384, both showing significant gene-gene interactions in BAMSE (**Fig 4**)**.** These results suggested that the epistatic signal with *NPS* was driven by genetic variants in the proximities of exon 3 and exon 4 in *NPSR1*.

## Discussion

Previous studies have shown that some polymorphic variants in the genes encoding *NPS* and *NPSR1* have functional effects in modifying peptide bioactivity or receptor signaling efficacy [26, 29]. However, no study has yet analyzed the functional outcomes of the different combinations of coding NPS and NPSR1 variants upon ligand/receptor interactions. This is of critical relevance, because coding SNPs such as *NPS* rs4751440 or *NPSR1* rs324981 are commonly observed in humans, and cells in any given individual will carry one out of nine alternatives of genotype combinations at these two loci. We examined the effects of the peptides NPS-Val(6) and NPS-Leu(6) on the signal transduction of NPSR1, expressed as two variants with different signaling efficacy (NPSR1-Ile(107) and NPSR1-Asn(107)). Our results clearly showed that the magnitude of NPSR1 signaling (as determined by reporter gene assays) and expression levels of downstream genes was dependent on the combination of NPS and NPSR1 variants, with the highest induction obtained upon stimulation of NPSR1-Ile(107) overexpressing cells with NPS-Val(6), and the lowest induction obtained with the combination of NPSR1-Asn(107) and NPS-Leu(6). Genetic association analyses further suggested epistasis between *NPS* and *NPSR1* variants with the most significant interactions being centered to the region of *NPSR1* rs324981 encoding for NPSR1-Ile(107) and NPSR1-Asn(107).

A key strength of this study is the use of robust *in vitr*o cell assays that allowed us to dissect the functional effects of coding NPS/NPSR1 variants on receptor signaling and downstream gene expression. Our cell assays confirmed that stimulation of cells transfected with either NPSR1-Asn(107) or NPSR-Ile(107) with the wildtype NPS-Val(6), induced CRE activity as described previously [26, 27]. Reinscheid *et al*. [26] have previously shown that upon stimulation with NPS-Val(6), stable HEK293 cell lines expressing NPSR1-Asn(107) and CRE-luciferase reporter gene displayed an induction of luciferase activity with a mean EC_50_ of 45 nM, which was roughly 10-fold lower than in this study (428 nM). For NPSR1-Ile (107), the stimulation with NPS-Val(6) produced an increase in reporter gene expression with an EC_50_ of 0.6 nM, which was 10-fold lower than what we observed (4.8 nM) [26]. The differences could be explained by the ∼20-fold lower magnitude of reporter gene induction in transiently transfected cells compared with stable clones [26]. In agreement with our study, the maximum of reporter gene expression in HEK293 cells transiently transfected with NPSR-Ile (107) was ∼2-fold higher than in cells expressing NPSR1-Asn(107), indicating an increase in agonist efficacy. Another previous study observed EC_50_ values of 37 nM and 586 nM for NPS-Val(6) and NPS-Leu(6), respectively, in CRE-luciferase activity 18 h after stimulation of HEK293 cells transiently transfected with NPSR1-Asn(107) [29]. Overall, our results were in line with these previous observations, and methodological issues, such as differences in NPS incubation time and transfection efficiency, might well explain the differences observed.

Our genetic analyses in the birth cohort BAMSE uncovered the association between *NPS* rs4751440 and the presence of asthma by 8 years. Another *NPS* SNP (rs10830123) showed borderline association with asthma, and formed a protective haplotype with *NPS* rs1931704 and *NPS* rs4751440. The protective effect of the minor allele C in *NPS* rs10830123 became significant only in the presence of two copies of rs324981 (TT) encoding NPSR1-Ile(107). In the replication cohort, *NPS* rs10830123 showed interaction with *NPSR1* rs17199659 which is in linkage disequilibrium with rs324981 and rs324384. Due to the LD, *NPS* rs10830123 can be used as a proxy for the effects of *NPS* rs4751440 (**Fig 4**). Although the latter had significant main effects on asthma risk and proved to be functional in the cell assays, a lower frequency of informative GC heterozygotes in the patient group could have influenced the power to detect gene-gene interactions with *NPSR1*. Indeed, 24% of non-asthmatic children were heterozygous for *NPS* rs4751440 and there was a low frequency of CC carriers in the cohorts with only 2% of children being homozygous (CC) for NPS-Leu(6). This was in line with previous findings suggesting that 22% of Europeans are heterozygous for *NPS* rs4751440 [29]. Nevertheless, it is consistent in BAMSE and MAGIC/ISAAC that the most significant interaction was centered to the region of *NPSR1* rs324981 encoding for NPSR1-Ile(107) and NPSR1-Asn(107). Altogether, our results supported the role of the NPS/NPSR1 pathway in asthma and the notion of a “combinatory effect”, in which the phenotype is driven by the interaction of several variants of moderate effects that coincide and affect a biological pathway [17].

In BAMSE, the *NPS* rs10830123 also showed significant interactions with two SNPs located in the upstream region of *NPSR1* (rs2125404 and rs2168890). These were in LD with *NPSR1* rs2530547 previously found to affect *NPSR1* mRNA levels in human leukocytes [27] and with three SNPs affecting *NPSR1* mRNA expression in cerebellum (rs2530549, rs2530550, and rs2530566) according to the Genotype-Tissue Expression database (http://gtexportal.org/home/gene/NPSR1). Furthermore, a bioinformatic analysis on the transcription binding sites that are created or destroyed by the five *NPSR1* SNPs implicated in the gene-gene interactions with *NPS* (**S3 Table**) revealed that the change of cytosine (C) to guanine (G) in *NPSR1* rs323922 is predicted to affect the binding site of V$NBRE/NBRE.01 related to the monomers of the nur subfamily of nuclear receptors (NR4A1-3, also known as nur77, nurr1, nor-1, **S4 Table)**.

Previous transcriptome studies in HEK293 cells [32, 33] and SH-SY5Y cells [36] overexpressing NPSR1-Ile(107) revealed downstream target genes with *NR4A1* and *FOS* being among the most affected transcription factors. In our cells assays, the magnitude of the mRNA expression of *NR4A1* was clearly dependent on the combination of NPS and NPSR1 variants **(Fig 2)**. *NR4A1* is expressed in lymphocytes upon stimulation and is anti-inflammatory [40–42]. *NR4A1* has been recently described as a key regulator of catecholamine production by macrophages linking sympathetic stress response and inflammation [43]. The hypothesis of a regulatory loop influenced by particular combinations of NPS/NPSR1 variants is further suggested by the up-regulation of the transcription factor *FOS* in cells expressing NPSR1-Ile(107) upon stimulation with NPS-Val(6) and the fact that the promoter region of *NPSR1* is known to contain FOS binding motifs [28]. The upregulation of *NR4A1*and *FOS* induced by the combination of NPS-Val(6) and NPSR1-Ile(107) is anticipated not only to interact in a feedback loop with their regulatory elements in *NPSR1* but also to regulate a number of other target genes including those involved in immune responses. For instance, the levels of serum pro-inflammatory cytokines could be activated by both central and peripheral administration of NPS in pigs [44], and NPS has shown direct effects on phagocytosis [45] [46] and chemotaxis [47] [48]. Further insights to immune responses are suggested by the upregulation of *CXCL2* and *CCL20* encoding chemokine receptors ligands **(Fig 2)**.

**Fig 5:**
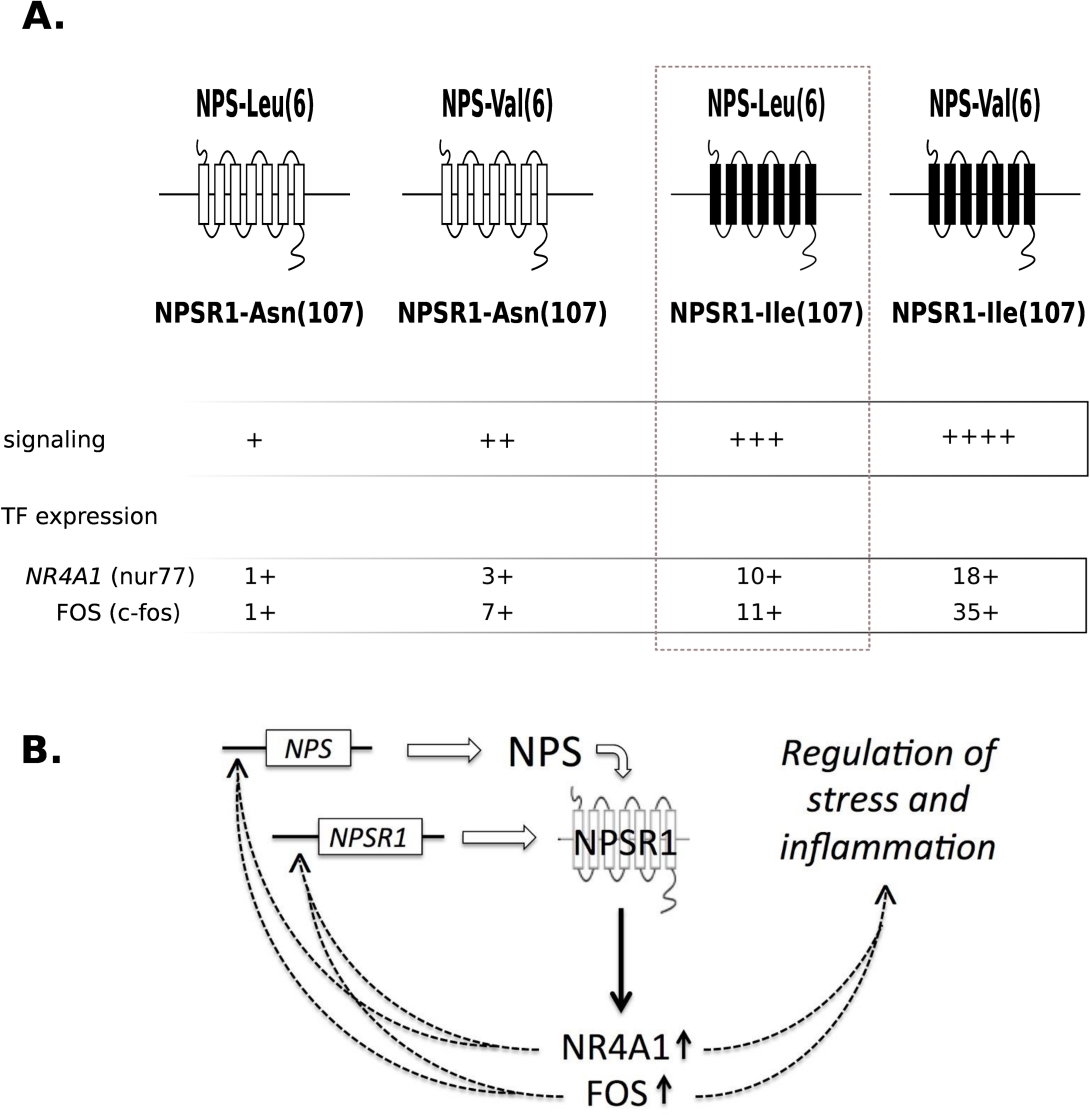
A schematic model on the functional crosstalk between NPS and NPSR1. **(A)** The non-synonymous NPS-Leu(6) substitution affects NPS bioactivity. Depending on the NPSR1-Asn(107) or NPSR1-Ile(107) genotype, binding of NPS can lead to strong or minimal signaling as well as differential expression of downstream target genes such as the transcription factors FOS and NR4A1. **(B)** Two hypothetical mechanisms in which the differential expression of FOS and NR4A1 can modulate the outcomes of the NPS/NPSR1 pathway. FOS and NR4A1 bind to their regulatory elements in *NPSR1* creating a feed-back loop that affect cell surface expression of NPSR1 and availability, and also, affect a number of other downstream genes that modulate neuroinflammation and ultimately asthma risk.

The findings of significant epistatic signals implicating coding and non-coding variants allowed us to suggest a model in which the divergent biological outcomes driven by coding variants in NPS and NPSR1 do occur at the level of ligand/receptor interactions but also implicate the effects of polymorphic variants in non-coding regions and the cross-talk of regulatory elements in diverse loci **(Fig 5)**. We must emphasize that the sample size analyzed in this study is underpowered to detect epistatic effects. However, the facts that the interaction signals in the two independent cohorts were narrowed to the same gene regions **(Fig 4)** and a recent meta-analysis on a larger dataset such as GABRIEL [49] confirmed the interaction signals that we originally identified in this population between *NPSR1* and *RORA* [17], suggest that the interactions between *NPS* and *NPSR1* may occur and are detectable at present resolution. Additional studies using larger sample sizes more suitable to test for epistasis are needed to confirm these findings.

In conclusion, our results supported the association of the NPS/NPSR1 pathway with asthma and provided a molecular framework on how the different NPS and NPSR1 variants interact in asthma. This study provides the basis for further investigations analyzing the interacting effects of NPS/NPSR1 variants in many other phenotypes and also support that polymorphic variants in *NPS* should be taken into account when analyzing the effects of *NPSR1* SNPs. Such gene-gene interactions as observed here may dilute or dampen association signals in genetic association studies.

## Materials and methods

### Ethics statement

All methods were carried out in accordance with relevant guidelines and regulations and the study followed the ethical principles for medical research stated in the Declaration of Helsinki. All of the experimental protocols were approved by the Swedish Regional Ethics Committee at Karolinska Institutet, Stockholm, Sweden in the BAMSE cohort (Dnr: 01–478 and 02–420) and by the Ethic Committee of the Bavarian Medical Council for MAGIC/ISAAC (approval nr. 01218). Written informed consent was obtained from parents/guardians on behalf of all child participants.

### Cell culture and NPS stimulations

Human embryonic kidney cells (HEK293) were cultured in MEM+GlutaMAX-1 medium (Gibco/Invitrogen) supplemented with 10% fetal calf serum (FCS) (Biosera), 1% sodium pyruvate, 1% non-essential amino acids and 1% penicillin/streptomycin (Gibco/Invitrogen). Cells were kept at 37°C in a humidified 5% CO_2_ incubator. The cells were transiently transfected (Lipofectamin™2000 Reagent, Invitrogen, Carlsbad, USA) with equal amounts of pCMV-*NPSR1-Ile(107)* or *NPSR1-Asn(107)* [27] in a ratio of 1:2 (DNA:Lipofectamin), and stimulated with NPS 24 h after transfection. The cells were stimulated with either 100 nM NPS-Val(6) (SFRNGVGTGMKKTSFQRAKS) or 100 nM NPS-Leu(6) (SFRNGLGTGMKKTSFQRAKS) (both from Proteogenix, France) for 1, 3, 6, and 24 h. The HEK293 cell line was chosen for ligand-receptor studies because it is a well-established model for addressing numerous questions in basic biology and is easy to transfect and amenable of stringent quantitative assessment.

The stable NPSR1-A-GFP expressing SH-SY5Y cells were engineered and cultured as described before [17]. For gene expression studies, the SH-SY5Y cells over-expressing NPSR1 were seeded at 0.5×10^6^ cells/ml to 6-well plates. After 24 h, fresh cell media was added, and the cells were incubated with a dilution series of either NPS-Val(6) or NPS-Leu(6) (0.0001–10 μM) for 3 h. The changes in gene expression were analyzed with real-time PCR (RT-PCR).

### Luciferase assays

For pharmacokinetic studies, HEK293 cells in 24-well plates were co-transfected with 700 ng of NPSR1 cDNA expression vectors specific for different coding variants and 50 ng each of cAMP response element (CRE) Firefly Luciferase reporter vector and CMV Renilla Luciferase reporter plasmid (purchased as pre-formulated mix from SABiosciences, Frederick, MD, USA). The day after transfection the cells were stimulated for 3 h with NPS at various concentrations (range 10 pM– 100 μM) before preparation of total cell lysates. In all reporter assays, Luciferase activity was measured using the Dual-Luciferase Reporter Assay System (Promega) according to the manufacturer's instructions, in a Microplate Reader Infinite 200 (Tecan, Männedorf, CH). Experiments were performed three times in triplicates, and Firefly Luciferase activity was expressed in arbitrary units relative to the cells transfected with the vector without NPS stimulation, after normalization for transfection efficiency based on values obtained for Renilla Luciferase. The concentration-response curve values for pEC_50_, and E_max_ were calculated using nonlinear regression analysis, and the data are presented as means ± SE. Statistical analysis was performed using one-way ANOVA followed by unpaired *t*-test for comparison between two groups. For the analyses, GraphPad Prism 5.01 (GraphPad Software, San Diego, CA) was used.

### Real-time RT-PCR

Total cellular RNA was isolated with the RNAeasy Mini Kit (Qiagen, Hilden, Germany). Reverse transcription was performed with TaqMan reverse transcription reagents (Applied Biosystems, Rotkreuz, Switzerland) using random hexamer primers according to the manufacturer's protocol.

The mRNA expression was measured with qRT-PCR using either SYBR Green (for *NR4A1*, *FOS*, *IER3*, and *EGR1*, the primer sequences are given in **S5 Table**) or TaqMAN assay (Hs00601975 for *CXCL2* Hs00601975, Hs01011368 for *CCL20*, Hs01036497 for *NPSR1* and Pre-Developed TaqMAN Assay Reagents, Part No. 4310884 for *GAPDH*; Applied Biosystems).

The PCR assays were performed in a total volume of 10 μl, using 7500 Fast Real-Time PCR system (Applied Biosystems) with the following reaction conditions: 50°C for 2 min and 94°C for 10 min; followed by 45 cycles of 92°C for 14 s and 1 min at 60°C. A dissociation stage was added to the SYBR Green reactions to confirm primer specificity. Results are shown as relative expression compared with unstimulated cells and *GAPDH* (Applied Biosystems) was used as an endogenous control. Samples were normalized to *GAPDH* (in SH-SY5Y cells) or to *GAPDH* and *NPSR1* in order to control for the efficiency of the transient transfections (in HEK293 cells). The variation in NPSR1 expression (Ct values) between cells transfected with the NPSR1-Asn(107) and NPSR1-Ile(107) was within one cycle. Expression levels of mRNA in each sample were determined by the comparative C_T_ method of relative quantification, and expressed in fold-changes relative to the chosen reference sample.

### Study populations

Genetic association analyses were conducted in the prospective Swedish birth-cohort BAMSE (Children Allergy Milieu Stockholm an Epidemiological study), which is an unselected, population-based Swedish birth cohort originally designed to assess risk factors for allergic diseases in childhood. This prospective study of 4,089 children has been described in detail elsewhere [38, 39]. At age 8, all children were invited to clinical testing and blood samples were obtained. After exclusion of samples with too little blood, incomplete questionnaire data, or lack of parental consent to genetic analysis there were 2033 samples with DNA extracted for genetic studies (of which 262 were asthmatics). All samples were analyzed coded. The study was approved by the Regional Ethical Committee of Karolinska Institutet, Stockholm, Sweden. Written informed consent was obtained from the parents and/or legal guardians.

Replication studies were carried out in 1454 children from the Multicenter Asthma Genetic in Childhood (MAGIC) study (mean age 11 y) and the cross sectional International Study of Asthma and Allergies in Childhood phase II (ISAAC II) study (age 9–11 y) [50–52]. From both populations, all samples which had been part of the first GWAS on asthma [50] and for which we had imputation data available were selected for this study. After quality control (QC), 1,454 samples (763 asthmatics and 683 controls) remained for analysis. All study methods were approved by the local ethics committees and written informed consent was obtained from all parents of children included in this study. To control for population stratification, all children were of German descent. Considering that previous studies have detected only marginal genetic differences between Swedes and Germans, and that GWAS data supported that within Europe, Germans and Swedes are most closely related [53], it can be assumed that the Swedish BAMSE and the German ISAAC/MAGICS were comparable.

### Phenotype assessment

Information on the clinical outcomes was obtained from questionnaires filled out by the parents, except for atopic sensitization which was assessed from plasma samples. In BAMSE, *Asthma ever up to 8 years* was defined as a physician-diagnosed asthma between 3 mo after birth and up to 8 y of age [54]. *Atopic sensitization* was considered present if a child had levels of allergen-specific IgE >0.35 kU/L to Phadiatop, a mixture of cat, dog, horse, birch, timothy, mugwort, *Dermatophagoides pteronyssinus*, and *Cladosporium* allergens at 8 y of age (ImmunoCAP, Thermo Fisher Scientific, Phadia AB, Uppsala, Sweden).

In MAGIC, the assessed variables included asthma and allergy, which were diagnosed by a pediatric pulmonologist according to clinical guidelines and objective measures such as lung function tests, clinical examination, and allergy testing [51, 55]. In ISAAC II, children were classified as having asthma if parents reported a physician’s diagnosis of asthma at least once, or of spastic or asthmatic bronchitis more than once in self-administered questionnaires [56, 57]. The populations of the Swedish BAMSE and the German ISAAC/MAGICS are assumed to be comparable because in both, the definition of the phenotype”asthma” was based on a physician-diagnosis and the age range of the children was comparable (8 years in BAMSE vs. 10 years in ISAAC/MAGICS) [58].

### SNP selection, genotyping and quality control

In BAMSE the *NPS* and *NPSR1* polymorphisms were genotyped by using the iPLEX chemistry on the SEQUENOM platform at the Mutation Analysis Facility (Karolinska Institutet), with the exception of rs4751440 which was genotyped by a TaqMan SNP Genotyping Assay in an ABI Prism 7500 Fast Sequence Detection System (Applied Biosystems) according to manufacturer's instructions. Primers for multiplex PCR and extension reactions were designed by the SpectroDesigner software (Sequenom GmbH, San Diego, CA, USA) and are available on request. Each assay was validated using 24 unrelated Caucasians and 3 CEPH DNA samples as well as 14 trios from the CEU population. Success rate for genotyping was 98.8%. In the MAGIC/ISAAC study, *NPS* and *NPSR1* SNPs were genotyped by the Illumina Sentrix HumanHap300 BeadChip as previously described [50]. The *NPS* gene spans a region of 3.3 kb on chromosome 10q26.2 whereas the *NPSR1* gene spans over ~220 kb of genomic DNA on chromosome 7p14. The SNPs included in this study were selected based on their effects on protein function (*e*.*g*. variants encoding for *NPS*-Val(6)Leu and NPSR1-Asn(107)Ile); previous reports of association with asthma in children from BAMSE [6, 17] and other populations [5–12]; and by their functional [27] and tagging properties [17, 19, 28]. We analyzed 3 *NPS* SNPs (rs1931704, rs10830123 and rs4751440 Val(6)Leu), 29 *NPSR1* SNPs in the BAMSE cohort [17, 19], and 23 *NPSR1* SNPs in MAGIC/ISAAC [50]. The discrepancy in the number of *NPSR1* SNPs was due to the fact that some SNPs genotyped in BAMSE were not present in the array. Genotype frequencies for all SNPs agreed with the expectations under Hardy-Weinberg equilibrium.

### Genetic association tests

Genetic association tests were conducted using Plink (version 1.07, http://pngu.mgh.harvard.edu/purcell/plink) using the commands—assoc and—model. The comparison of allele frequencies between cases and controls were done by χ^2^ and Fisher's exact test. Full model associations were evaluated using the Cochran-Armitage trend test, and genotypic, dominant and recessive models. The genotypic and dominant/recessive tests were only conducted if there was a minimum number of five observations per cell either in the 2-by-3 or 2-by-2 tables. Since we tested 32 individual SNPs it was expected at least 1.6 SNPs to be significant (p < 0.05) due to chance. Epistasis was evaluated using the—epistasis command in PLINK. All pairwise SNP combinations were tested in the case-control datasets. Allelic by allelic epistasis between *NPS* and *NPSR1* SNPs were calculated by logistic regression. The allele dosage of each SNP (A and B) was modeled in the form of *Y ~ β0 + β1*.*A + β2*.*B + β3*.*AB + e* and the test for interaction based on the coefficient *β3*. In the equation, *Y* represents the presence of asthma as a function of the intercept (*β0*) and the coeffients of the different alleles, and *e* the residual error.

## Supporting information

S1 Table Genetic association of NPS polymorphisms with asthma at age 8 years in the BAMSE cohort. (DOCX)

S2 Table Genetic association of NPSR1 polymorphisms with asthma at age 8 years in the BAMSE cohort. (XLSX)

S3 Table Interactions between *NPS* and *NPSR1* on the risk of asthma under the multiplicative model. (DOCX)

S4 Table Predicted changes in transcription factor binding sites encoded by the SNPs implicated in the epistasis between NPS and NPSR1 as determined by the Genomatix Software Suite. (XLS)

S5 Table Primers used for qPCR analysis in the human cell lines and mice experiments. (DOCX)
