# Supplementary material for: Neuropeptide S (NPS) variants modify the signaling and risk effects of NPS Receptor 1 (NPSR1) variants in asthma
Source: PLoS One. 2017 May 2;12(5):e0176568. doi: 10.1371/journal.pone.0176568 (PMC5413018; doi:10.1371/journal.pone.0176568)
Supplement: S1 Table — (DOCX) [file pone.0176568.s001.docx]

**S1 Table.** Genetic association between *NPS* polymorphisms with asthma at age 8 years in the BAMSE cohort

| CHR | SNP | A1 | A2 | TEST* | AFF | UNAFF | CHISQ | DF | P |
| --- | --- | --- | --- | --- | --- | --- | --- | --- | --- |
| 10 | rs1931704 | A | G | GENO | 15/91/180 | 99/631/911 | 5.486 | 2 | 0.06 |
| **10** | **rs1931704** | **A** | **G** | **TREND** | **121/451** | **829/2453** | **4.458** | **1** | **0.03** |
| **10** | **rs1931704** | **A** | **G** | **ALLELIC** | **121/451** | **829/2453** | **4.42** | **1** | **0.03** |
| **10** | **rs1931704** | **A** | **G** | **DOM** | **106/180** | **730/911** | **5.462** | **1** | **0.01** |
| 10 | rs1931704 | A | G | REC | 15/271 | 99/1542 | 0.2718 | 1 | 0.6 |
| 10 | rs10830123 | C | G | GENO | 6/57/215 | 36/406/1148 | 3.284 | 2 | 0.1 |
| 10 | rs10830123 | C | G | TREND | 69/487 | 478/2702 | 2.58 | 1 | 0.1 |
| 10 | rs10830123 | C | G | ALLELIC | 69/487 | 478/2702 | 2.602 | 1 | 0.1 |
| 10 | rs10830123 | C | G | DOM | 63/215 | 442/1148 | 3.165 | 1 | 0.07 |
| 10 | rs10830123 | C | G | REC | 6/272 | 36/1554 | 0.01207 | 1 | 0.9 |
| **10** | **rs4751440** | **C** | **G** | **GENO** | **6/50/231** | **30/404/1217** | **6.786** | **2** | **0.03** |
| **10** | **rs4751440** | **C** | **G** | **TREND** | **62/512** | **464/2838** | **4.4** | **1** | **0.03** |
| **10** | **rs4751440** | **C** | **G** | **ALLELIC** | **62/512** | **464/2838** | **4.405** | **1** | **0.03** |
| **10** | **rs4751440** | **C** | **G** | **DOM** | **56/231** | **434/1217** | **5.941** | **1** | **0.01** |
| 10 | rs4751440 | C | G | REC | 6/281 | 30/1621 | 0.1003 | 1 | 0.7 |

The TEST columns explains which test was used (GENO= basic genotypic, TREND=additive test, DOM=dominant test and REC=recessive test).
