# Supplementary material for: Neuropeptide S (NPS) variants modify the signaling and risk effects of NPS Receptor 1 (NPSR1) variants in asthma
Source: PLoS One. 2017 May 2;12(5):e0176568. doi: 10.1371/journal.pone.0176568 (PMC5413018; doi:10.1371/journal.pone.0176568)
Supplement: S3 Table — (DOCX) [file pone.0176568.s003.docx]

**S3 Table.** Interactions between *NPS* and *NPSR1* on the risk of asthma under the multiplicative model

| **CHR** | **SNP1**  ***NPSR1*** | **coordinate** | | **CHR** | **SNP2**  ***NPS*** | **Population** | **STAT** | **Interaction OR (95%CI)** | **p-value** |
| --- | --- | --- | --- | --- | --- | --- | --- | --- | --- |
| 7 | rs2125404 | | 34657776 | 10 | rs10830123 | BAMSE | 4.03 | 0.47 (0.22-0.98) | 0.04 |
| 7 | rs2168890 | | 34657860 | 10 | rs10830123 | BAMSE | 4.71 | 0.42 (0.19-0.92) | 0.02 |
| 7 | rs323922 | | 34709170 | 10 | rs10830123 | BAMSE | 4.62 | 0.64 (0.42-0.96) | 0.03 |
| 7 | rs324384 | | 34742415 | 10 | rs10830123 | BAMSE | 6.11 | 1.62 (1.10-2.39) | 0.01 |
| 7 | rs324981  Asn(107)Ile | | 34778501 | 10 | rs10830123 | BAMSE | 6.79 | 0.59 (0.39-0.87) | 0.009 |
| 7 | rs17199659 | | 34812217 | 10 | rs10830123 | MAGIC/ISAAC | 7.93 | 1.78 (1.19-2.67) | 0.004 |
