# Supplementary material for: Neuropeptide S (NPS) variants modify the signaling and risk effects of NPS Receptor 1 (NPSR1) variants in asthma
Source: PLoS One. 2017 May 2;12(5):e0176568. doi: 10.1371/journal.pone.0176568 (PMC5413018; doi:10.1371/journal.pone.0176568)
Supplement: S5 Table — (DOCX) [file pone.0176568.s005.docx]

**S5 Table.** Primers used for qPCR analysis in the human cell lines and mice experiments

| Gene | Accession | 5' Primer | 3' Primer | Length |
| --- | --- | --- | --- | --- |
| *FOS* | NM_005252 | TGACTGATACACTCCAAGCGGA | CCTTCAGCAGGTTGGCAATC | 84 |
| *NR4A1* | NM_001202233 | GAAACCGCTGCCAGTTCTG | TCTGTTCGGACAACTTCCTTCA | 73 |
| *IER3* | NM_003897.3 | GCAGCCGCAGGGTTCTC | ACGATGGTGAGCAGCAGAAA | 100 |
| *EGR1* | NM_001199880.1 | CACCTGACCGCAGAGTCTTT | TGGGGTAACTGGTCTCCACC | 73 |
| *GAPDH* | NM_002046 | AACAGCGACACCCATCCTC | CATACCAGGAAATGAGCTTGACAA | 81 |
